# NLRP3 Inflammasome and IL-1–Mediated Inflammation in Human Carotid Atherosclerosis: A Systematic Review of Endarterectomy-Based Evidence

**DOI:** 10.3390/medsci14020280

**Published:** 2026-05-31

**Authors:** David Mendonça-Soares, Antónia Rocha-Melo-Sousa, Mohammed Shahat, Cármen Tavares, Lina Carvalho, Vitor Sá-Martins, Manuel Neiva-Sousa, Mariana Fragão-Marques, João Rocha Neves

**Affiliations:** 1 Faculty of Medicine, University of Porto, 4200-319 Porto, Portugal; 2 MEDCIDS—Department of Community Medicine, Information and Health Decision Sciences, Faculty of Medicine, University of Porto, 4200-319 Porto, Portugal; 3 Department of Vascular and Endovascular Surgery, Assiut University Hospitals, Assiut 2074020, Egypt; 4 Nova Medical School, 1169-056 Lisboa, Portugal; 5 Institute of Anatomical and Molecular Pathology, Faculty of Medicine, University of Coimbra, 3000-370 Coimbra, Portugal; 6 Functional Unit of Maxillofacial Surgery, Unidade Local de Saúde do Alto Ave (ULSAAVE), 4835-044 Guimarães, Portugal; 7 School of Medicine and Biomedical Sciences, Universidade Fernando Pessoa, 4420-096 Gondomar, Portugal; 8 Department of Imuno-Physiology and Pharmacology, Instituto de Ciências Biomédicas Abel Salazar (ICBAS), Universidade do Porto, 4050-313 Porto, Portugal; 9 Unit of Anatomy, Department of Biomedicine, Faculty of Medicine, University of Porto, 4200-319 Porto, Portugal; 10 RISE-Health, Departamento de Biomedicina, Faculdade de Medicina, Universidade do Porto, 4200-319 Porto, Portugal; 11 Department of Vascular Surgery, Unidade Local de Saúde do Alto Ave (ULSAAVE), 4835-044 Guimarães, Portugal

**Keywords:** carotid artery diseases, atherosclerosis, cytokines, biomarkers, prognosis, risk factors

## Abstract

**Background**: Carotid atherosclerosis and plaque instability are major drivers of ischemic stroke. The NLRP3 inflammasome and interleukin-1 (IL-1) pathway are recognized as key upstream regulators of atherogenesis. This systematic review aims to synthesize the available evidence regarding the impact of NLRP3 and IL-1-mediated inflammation on plaque vulnerability and clinical outcomes in patients undergoing carotid endarterectomy (CEA). **Materials and Methods**: A systematic search was performed relying on MEDLINE, Scopus, and Web of Science for studies assessing NLRP3 inflammasome and IL-1-mediated biomarkers in adult patients undergoing CEA. Data extraction and risk-of-bias evaluation were independently performed using the National Heart, Lung, and Blood Institute (NHLBI) Quality Assessment Tool. Due to substantial methodological heterogeneity, a narrative synthesis was conducted. **Results**: Sixteen studies involving 1677 participants were included. Evidence demonstrates that NLRP3 inflammasome components (NLRP3, ASC, Caspase-1) and IL-1 family cytokines (IL-1β, IL-18) are consistently elevated in unstable plaques and in symptomatic patients compared to asymptomatic counterparts. These markers were associated with histological features of instability, such as intraplaque haemorrhage, large lipid cores and extensive macrophage infiltration. While some data suggest a link between these biomarkers and Major Adverse Cardiovascular Events (MACE), most studies were limited by a lack of adjusted multivariable modelling and an overall unclear risk of bias. **Conclusions**: NLRP3/IL-1/-mediated inflammation is a promising biomarker axis for carotid plaque vulnerability and symptomatic disease. However, small, heterogeneous cohorts and limited adjusted analyses highlight the need for larger, well-designed studies to refine risk stratification and guide targeted anti-inflammatory strategies in carotid atherosclerosis.

## 1. Introduction

Carotid atherosclerosis is associated with severe clinical complications, most notably ischemic stroke and transient ischemic attack [1]. In fact, extracranial internal carotid stenosis is a major contributor to cerebrovascular morbidity, accounting for approximately 10% to 15% of all ischemic strokes globally [2]. Plaque instability and inflammatory activity contribute to embolization risk independently of luminal stenosis severity [1]. Carotid endarterectomy (CEA) remains a cornerstone intervention for selected patients with significant stenosis, yet perioperative and long-term adverse outcomes, including: stroke, myocardial infarction, restenosis, and mortality continue to occur [1]. Increasing evidence suggests that inflammatory biomarker profiles within plaque tissue and circulation may be associated with plaque vulnerability and clinical outcomes, but reported findings vary substantially across studies [3].

Human carotid endarterectomy specimens provide a unique translational platform to directly investigate plaque biology through molecular, histological, and immunological analyses [4]. Multiple studies have measured interleukin-1 (IL-1)-related cytokines, NLRP3 inflammasome components, and associated immune markers in both carotid plaque tissue and peripheral blood, using diverse laboratory techniques and statistical models [5]. However, methodological heterogeneity and variable reporting limit cross-study interpretation and translational integration [3].

Among inflammatory mechanisms involved in atherogenesis, the IL-1 pathway has emerged as a major upstream regulator [5]. IL-1 signaling promotes endothelial activation, leukocyte recruitment, smooth muscle cell modulation, and matrix degradation [5]. Activation of the NLRP3 inflammasome further amplifies this response by enabling caspase-1–dependent maturation and release of IL-1β and IL-18 [5]. Cholesterol crystals, oxidative stress, and cellular damage signals present within plaques are known triggers of NLRP3 activation, linking metabolic stress to inflammatory amplification [5]. While experimental and translational studies strongly support the relevance of this pathway, human evidence derived specifically from plaque tissue and its relationship to circulating biomarkers and clinical outcomes is dispersed across heterogeneous, often underpowered investigations [4].

The objective of this systematic review is to synthesize evidence from human CEA investigations evaluating IL-1 and NLRP3-related biomarkers, including measurement techniques, quantitative findings, adjusted analytical models, and methodological quality assessed through structured risk-of-bias evaluation. Furthermore, standardised pathological evaluation of endarterectomy specimens, informed by the evidence synthesised here, may provide actionable insights to guide postoperative patient follow-up and risk stratification.

## 2. Methods

This systematic review was conducted in accordance with the Preferred Reporting Items for a Systematic Review and Meta-analysis (PRISMA) Statement and the AMSTAR -2 critical appraisal tool [6,7]. Institutional review board approval was not obtained due to the nature of this study. The review protocol has been prospectively registered at Prospero (reference: CRD420251233138).

### 2.1. Selection Criteria

Inclusion criteria consisted of all original research studies conducted in humans that evaluated the association between NLRP3 inflammasome and IL-1-related inflammatory biomarkers and outcomes in patients undergoing CEA. Eligible studies met all of the following criteria: enrolled adult patients undergoing CEA for symptomatic or asymptomatic internal carotid artery stenosis; measured biomarkers related to the NLRP3 inflammasome pathway and/or IL-1-mediated inflammation in systemic circulation and/or carotid plaque tissue; and reported associations between these biomarkers and at least one of the following: plaque vulnerability characteristics, perioperative outcomes, or long-term major adverse cardiovascular events (MACE). Animal or other non-human studies, case reports, case series with fewer than 20 patients, narrative reviews, systematic reviews, editorials, conference abstracts without extractable data, and studies in which relevant data could not be reliably extracted despite attempts to contact authors were excluded. No restrictions were applied regarding publication year or language; articles in languages other than English were screened with translation support as needed.

### 2.2. Search Strategy

A systematic search was performed in three databases: PubMed, Scopus, and Web of Science, in December 2025. The query and keywords are shown in Supplementary Table S1. Additionally, the references of the included primary studies and relevant available systematic reviews were screened to identify any further articles of potential interest.

**Table 1:** Study characteristics.

Author	Journal	Publication Year	Study Design	Study Center	Recruitment Time	Sample Size (Patients)	No. CEA	GRADE
[8]	European Journal of Vascular and Endovascular Surgery	2000	Prospective comparative study during CEA (shunt vs. no shunt based on ICBP)	St Vincent’s Hospital/Garvan Institute of Medical Research	NA	20	20	NA
[9]	PLOS ONE	2016	Cross-sectional	University of Bari	NA	41	41	NA
[10]	PLOS ONE	2014	Prospective study: CAS patients stratified by MRI plaque vulnerability	Mie University Hospital	2009 to 2012	58	17	NA
[11]	Journal of Stroke and Cerebrovascular Diseases	2015	Cross-sectional	NA	NA	50	30	NA
[12]	Atherosclerosis	2014	Cross-sectional	Lund University/Malmö (Sweden)	NA	200	200	NA
[13]	Atherosclerosis	2023	Retrospective cohort	Multicenter (Italy/Belgium)	NA	645	645	NA
[14]	Atherosclerosis	2012	Ex vivo/in vitro	Karolinska Institutet	NA	164	164	NA
[15]	Scientific Reports	2020	Prospective cohort	Single-center (vascular surgery/neurology unit)	2017-07 to 2018-06	46	46	NA
[16]	Annals of Vascular Surgery	2025	Prospective cohort	Royal Liverpool and Broadgreen University Hospital NHS Trust	NA	75	13	NA
[17]	Clinical and Experimental Immunology	2008	Observational cohort	Istituto Superiore di Sanità	NA	106	67	NA
[18]	Cytokine	2013	Cross-sectional	UNICAMP (Campinas) departments of pathology/clinical pathology/surgery	NA	57	57	NA
[19]	International Journal of Molecular Medicine	2013	Cross-sectional	Univ. of Heidelberg	NA	20	20	NA
[20]	Iranian Journal of Public Health	2023	Case–control	The Second Hospital of Dalian Medical University, China	2013-01 to 2019-12	100	60	NA
[21]	Circulation	2009	Ex vivo/in vitro	NA	NA	NA	NA	NA
[22]	Antioxidants and Redox Signaling	2021	Cross-sectional/Ex vivo	University of Debrecen	NA	NA	NA	NA
[23]	Archives of Medical Science	2013	Case–control	Medical Centre of Silesia	NA	95	65	NA

Legend: CAS—carotid artery stenting; CEA—carotid endarterectomy; NA—not available.

### 2.3. Study Selection and Data Extraction

After duplicate removal, two authors (DS and MARM) independently participated in study selection; any disagreements were resolved by a third author (JRN). First, studies were selected based on title and abstract, and the remaining studies were eligible for full-text assessment. Efforts were made to contact the authors to obtain the full texts that were not publicly available. The selected studies were carefully revised to avoid repeated populations.

Data from included studies were independently extracted by two authors (DS and MARM). Data were extracted using a fit-for-purpose form on the year of publication, country, centre of recruitment, study design, recruitment time, number of participants undergoing carotid endarterectomy, participants’ age and gender distribution, frequency of cardiovascular comorbidities, and carotid symptomatic status.

### 2.4. Assessment of Study Quality

Concerning qualitative assessment, the National Heart, Lung, and Blood Institute (NHLBI) Study Quality Assessment Tool was used for observational cohort, cross-sectional and case–control studies (2021) [24]. This assessment was independently performed by two authors (DS and MARM), and when disagreements arose, decisions were made by mutual consensus following a third-party review (JRN). The quality of evidence for the included articles was evaluated using the Grading of Recommendations, Assessment, Development, and Evaluation (GRADE) approach. Articles were classified into four quality levels (high, moderate, low, and very low) [25].

### 2.5. Quantitative Synthesis

A quantitative meta-analysis was not feasible due to substantial methodological heterogeneity across studies, including inconsistencies in outcome definitions, follow-up periods, and reporting formats. As a result, the association of IL-1 and NLRP3 measurements with endarterectomy-related postoperative outcomes was synthesized narratively. Study characteristics, event rates, and the associations of biomarker expression levels with both features of plaque vulnerability and adverse clinical outcome, were compared descriptively, and sources of variability were examined qualitatively to provide an integrated interpretation of the available evidence.

Pre-specified covariates extracted for each study included publication year, mean age of participants, percentage of male patients, prevalence of arterial hypertension, dyslipidaemia, diabetes mellitus, and coronary artery disease (CAD), percentage of symptomatic carotid stenosis, and percentage of patients receiving antiplatelet therapy.

## 3. Results

### 3.1. Search Results

**Figure 1:**
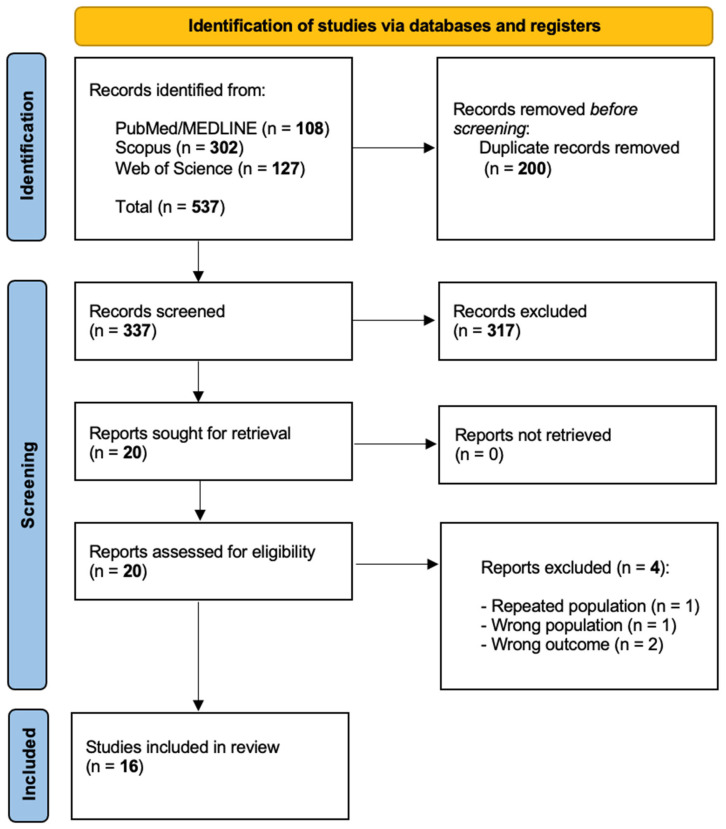
PRISMA Flow chart.

After the database search and duplicate exclusion, 337 studies were screened. Of the 337 studies selected by title and abstract, 317 were excluded. Twenty studies were eligible for full-text assessment; four were excluded during this process. Comprehensive reasons for exclusion upon full-text assessment were: repeated patient population (N = 1), wrong patient population (N = 1) and wrong outcome (N = 2). Thus, a total of 16 published articles were included in this systematic review. (Figure 1) [8,9,10,11,12,13,14,15,16,17,18,19,20,21,22,23].

### 3.2. Description of Studies

All studies included in this systematic review were observational or cross-sectional, with 1 being retrospective (Table 1) [13]. The included publications were performed in multiple countries within 3 continents: 12 from Europe (8, 9, 12-17, 19, 21-23), 3 from Asia [10,11,20] and 1 from South America [18]. A total of 1677 patients were assessed across the fourteen cohorts with a mean age of approximately 67 years (range 64–74 years) and 69% being male (n = 1155). Two additional mechanistic ex vivo/in vitro studies focusing primarily on plaque tissue and cell culture [21,22] were included qualitatively but were not counted towards the aggregate patient sample size. The number of participants per study ranged from 20 [18,21] to 645 patients [13]. Demographics and comorbidities of the populations included in the studies were gathered and are available in Table 2.

Most of the included studies comprised mixed cohorts of symptomatic and asymptomatic patients, enabling comparative analyses of plaque vulnerability and clinical presentation. Cardiovascular risk factors were highly prevalent among study participants: hypertension ranged from 70 to 90% across studies, diabetes mellitus from 15 to 50%, dyslipidemia from 45 to 80%, CAD from 13 to 50%, and active smoking from 20 to 60%.

### 3.3. Main Findings

#### 3.3.1. NLRP3/IL-1 in Atherosclerotic Plaques

NLRP3 inflammasome components and IL-1 family cytokines are consistently detected in atherosclerotic plaques and are significantly associated with plaque instability and symptomatic status [10,11,12,19,20,21]. NLRP3 inflammasome activation in human carotid plaques was characterized by the co-expression of core pathway components (NLRP3, ASC, Caspase-1), with significantly higher expression in morphologically unstable plaques [11,20]. Histologically, these vulnerable lesions were defined by the presence of a large lipid-necrotic core, a thin fibrous cap, and intraplaque haemorrhage. Within these lesions, inflammasome components were localized predominantly in the cytoplasm of macrophages and foam cells and were associated with cholesterol crystal clefts inside and outside cells [11,20]. ELISA also showed that the serum levels of the cytokines IL-1β and IL-18 were higher in the CEA group compared to controls, which suggests that serum levels of these cytokines may be useful predictors of atherosclerosis [11] (Table 3).

#### 3.3.2. Mechanistic Insights from Ex Vivo and In Vitro Investigations

To complement the clinical and translational evidence, three studies using human carotid plaque-derived cells or tissue ex vivo were included to characterise the mechanistic underpinnings of NLRP3/IL-1 activation. IL-1β emerged as a central effector of this pathway within the plaque tissue. IL-1β mRNA and protein levels were significantly higher in plaques from symptomatic patients (defined as those presenting with a recent history of ipsilateral ischemic stroke, transient ischemic attack (TIA), or amaurosis fugax) than in those of asymptomatic individuals [11,12,19,21]. Furthermore, IL-1β expression correlated directly with the severity of histological markers of vulnerability, including high macrophage density and matrix degradation [19]. Ex vivo work Monaco et al., using human carotid atheroma cells, highlighted the critical role of this upstream innate immune signalling: blockade of the shared IL-1/TLR adaptor MyD88 with a dominant-negative construct (MyD88DN), or direct antagonism of the IL-1 receptor with IL-1 receptor antagonist (IL-1Ra), significantly reduced the production of downstream pro-inflammatory mediators, particularly IL-6, demonstrating that IL-1-driven signalling is required for sustained plaque inflammation and matrix degradation [21]. Similarly, IL-18, another caspase-1-dependent cytokine, showed higher levels in patients with unstable plaques and symptomatic carotid disease, further highlighting the role of the inflammasome in driving the clinical transition from silent atherosclerosis to acute cerebrovascular events [11].

#### 3.3.3. Systemic Biomarkers

Systemic assessment of this pathway highlights its potential for the detection of circulating biomarkers. Xie et al. showed that NLRP3 and caspase-1 expression in peripheral blood mononuclear cells (PBMCs) correlated positively with a standardized Modified Plaque Vulnerability Risk Score (MPVRS) [20]. Specifically, systemic expression of these inflammasome components was significantly higher in patients with highly unstable plaques (MPVRS > 4) compared to those with stable lesions.

Supporting the link between blood biomarkers and imaging, Puz et al. evaluated preoperative serum and found that circulating IL-1β, IL-6, and TNF-α were significantly elevated in patients with hypoechoic (unstable) plaques as detected by ultrasound, whereas anti-inflammatory IL-10 was markedly lower [23,26].

Flow-cytometric data from Profumo et al. further demonstrated that this systemic inflammatory state is not merely transient [17]. The authors found persistently higher intracellular IL-1β (alongside other pro-inflammatory cytokines) in circulating monocytes from symptomatic patients at baseline, which remained significantly elevated at 1, 3, and even 6 months post-CEA, indicating a chronic systemic activation pattern. In systemic network analyses, Stauss et al. revealed that plasma IL-1β levels were explicitly higher in symptomatic stenosis (0.18 ± 0.05 vs. 0.12 ± 0.03 pg/mL, *p* < 0.05), and circulating IL-18 clustered with other pro-inflammatory cytokines in symptomatic stenosis, suggesting a coordinated systemic activation of the inflammasome-related axis [15].

Taken together, the systemic biomarker data converge on a consistent pattern: circulating and intracellular markers of NLRP3/IL-1 activation track plaque vulnerability across different biological compartments (peripheral blood mononuclear cells and plasma) and across methodologically distinct approaches [20,23]. Notably, this systemic activation appears to persist beyond the surgical event itself, as Profumo et al. demonstrated elevated monocytic IL-1β up to six months post-CEA, suggesting that CEA removes the local trigger but not the underlying systemic inflammatory state [17]. Whether this residual activation represents a therapeutic target or merely a marker of systemic atherosclerotic burden remains unanswered by the available evidence.

#### 3.3.4. Short-Term Outcomes

Evidence linking the NLRP3/IL-1 axis specifically to short-term or perioperative clinical events remains limited (Table 4), but pathophysiological shifts during the perioperative period have been well documented. Ahmed et al. prospectively evaluated 75 patients undergoing non-cardiac vascular surgery, including a carotid endarterectomy subgroup of 13 patients, and found that elevated baseline and peak postoperative serum IL-1β, IL-6, and CRP levels were associated with a 17.3% rate of 30-day major adverse cardiac events [16]. However, the small CEA subgroup limits carotid-specific inference. At the systemic level, IL-1β levels increased after carotid clamping and declamping, supporting a direct link between surgical ischaemia–reperfusion injury and IL-1 release into the cerebral circulation during the immediate short-term perioperative setting [8].

#### 3.3.5. Long-Term Outcomes

A small number of studies successfully linked this inflammatory pathway to long-term hard clinical outcomes, with results consistent with a potential prognostic impact, as shown in Table 5. In a large retrospective cohort of 645 patients, Marfella et al. compared 159 patients on PCSK9 inhibitor monotherapy with 486 patients receiving other lipid-lowering drugs (all on statins, with or without ezetimibe), and found that PCSK9 inhibitor use was associated with lower intraplaque expression of NLRP3, caspase-1, IL-1β, TNF-α, and NF-κB, higher levels of SIRT3 and collagen, and reduced MMP-9, consistent with a more stable plaque phenotype; over a mean follow-up of 678 ± 120 days, the PCSK9 inhibitor group experienced fewer composite major adverse cardiovascular events (non-fatal myocardial infarction, non-fatal stroke, and all-cause mortality) than the other lipid-lowering drugs group (10 vs. 82 events), corresponding to an adjusted hazard ratio of 0.262 (95% CI: 0.131–0.524), an effect that persisted even in LDL-cholesterol–matched subgroups with LDL-C < 100 mg/dL, suggesting a potential mechanistic link, though causality cannot be inferred from this study design [13]. Whether pharmacological modulation of the NLRP3/IL-1 axis through PCSK9 inhibition translates into clinically meaningful benefit in the CEA population remains to be established in prospective, interventional studies. In another cohort, Xie et al. evaluated 60 patients undergoing CEA for carotid atherosclerosis and found that increased PBMC expression of NLRP3 inflammasome components was associated with plaque instability; during a median follow-up of 33.99 months, composite all-cause mortality and non-fatal cardiovascular/cerebrovascular events occurred in 21.1% of patients with unstable plaques versus 13.6% of those with stable plaques [20].

#### 3.3.6. Upstream and Downstream Relationships

The activation of the NLRP3/IL-1 axis is driven by specific upstream pathophysiological triggers within the plaque. Mechanistic investigations demonstrated that the Toll-Like Receptor (TLR) pathway acts as a primary upstream regulator. Specifically, blocking the MyD88 adapter protein in human atheroma cells significantly reduced IL-1β production and its downstream inflammatory mediators [21]. Furthermore, intraplaque haemorrhage itself appears to act as an important upstream catalyst of NLRP3/IL-1-related inflammation. Potor et al. demonstrated that oxidized hemoglobin accumulates predominantly in complicated, haemorrhagic carotid plaques, lesions typically regarded as advanced unstable plaques, and directly activates human macrophages, significantly upregulating IL-1β and TNF-α expression [22].

Once triggered by these upstream mechanisms, downstream inflammatory markers consistently mirror this activation rather than act as isolated signals. The classic downstream effectors IL-6 and TNF-α were frequently elevated in patients with unstable or symptomatic plaques and served as central nodes in systemic cytokine networks [12,15,20,23]. Similarly, acute-phase reactants driven by the IL-1/IL-6 axis, such as CRP, PTX3, and fibrinogen, were significantly higher in hypoechoic, ulcerated, or hemorrhagic plaques. In line with these findings, Marzullo et al. reported that ST2L, the membrane receptor for the IL-1 family cytokine IL-33, showed stronger immunohistochemical expression on macrophages in symptomatic compared to asymptomatic carotid plaques (77.7% vs. 39.1%; *p* = 0.015), particularly in AHA type VI lesions and neoangiogenic vessel endothelium at the plaque shoulder, suggesting that activation of the ST2L/IL-33 axis may accompany the broader IL-1-driven inflammatory milieu in vulnerable plaques [9]. Crucially, these downstream markers co-localized directly with macrophage-rich, IL-1-positive regions, directly linking upstream inflammasome activation to downstream matrix degradation and plaque vulnerability [10,19,22,23]. In the systemic circulation, Profumo et al. found that circulating monocytes co-expressing intracellular IL-1β, IL-6, and IFN-γ were significantly elevated in patients with a history of stroke, indicating coordinated systemic activation of this entire upstream-downstream axis [17].

### 3.4. Studies Quality

**Figure 2:**
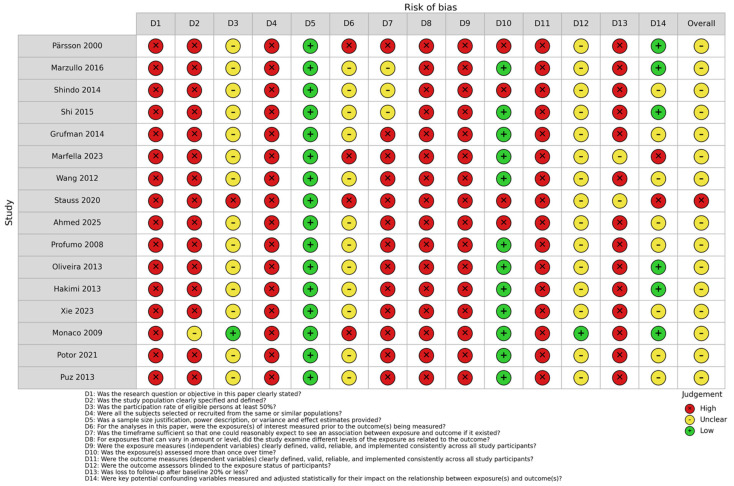
Overall risk-of-bias judgment for included studies.

Overall, the methodological quality of the evidence was limited. Most of the included studies were judged to have an overall “Unclear” risk of bias, while one study was classified as having “High” risk of bias, and none achieved an overall “Low” risk rating. Individual study ratings are presented in Figure 2.

**Figure 3:**
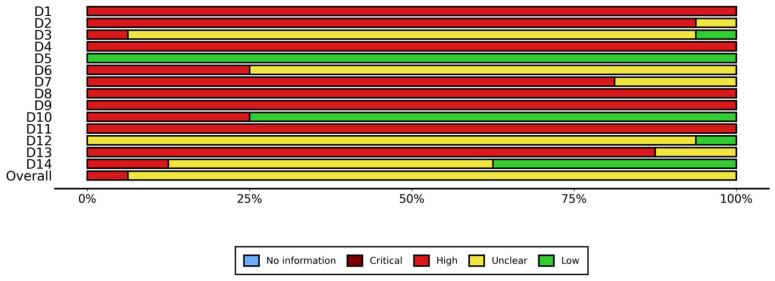
Risk-of-bias assessment across individual NHLBI quality domains.

Systematic evaluation across the 14 NHLBI quality criteria (Figure 3) revealed recurrent weaknesses in several domains. Items related to the definition and measurement of exposures (inflammatory biomarkers) and outcomes, as well as to the handling of potential confounders, were most frequently rated as unclear or high risk of bias. In many cohorts, biomarker assessment methods were not fully standardized or adequately described, and outcome definitions and adjudication procedures were not consistently specified.

The most critical methodological deficiency was the lack of confounding adjustment. In 14 out of 16 studies, associations between biomarkers and plaque vulnerability or clinical events were explored using univariable analyses or descriptive comparisons only, without multivariable modelling. Only two studies incorporated adjusted analyses: one study used multivariable Cox regression, including clinical covariates, to evaluate the association between plaque IL-1β, lipid-lowering therapy, and long-term MACE [13], and another used logistic regression with adjustment for stenosis severity and baseline vascular risk to examine cytokine networks [15]. All remaining cohorts did not account for key confounders such as age, sex, cardiovascular risk factors, medical therapy, or degree of stenosis, which substantially limits causal inference.

### 3.5. Publication Bias

Because included studies were highly heterogeneous in study design, biomarker methodologies (immunohistochemistry, ELISA, PCR, Western blot, flow cytometry), tissue sources (plaque tissue, peripheral blood, PBMCs), and reported outcomes (biomarker expression, plaque vulnerability, perioperative events, long-term MACE), no quantitative pooling or meta-analysis was performed. Therefore, publication bias was not formally assessed using funnel plots. The narrative synthesis approach was deemed most appropriate given the substantial methodological and clinical diversity across included investigations.

## 4. Discussion

The evaluation of human carotid endarterectomy cohorts provides a unique opportunity to investigate both local plaque tissue and systemic circulating biomarkers, shedding light on the role of the NLRP3 inflammasome and IL-1–mediated inflammation in carotid atherosclerosis, plaque vulnerability, and postoperative outcomes. Across 16 observational cohorts, a consistent association was observed between local and systemic inflammatory biomarker expression and features of plaque instability, whereas evidence linking these biomarkers to perioperative or long-term major adverse cardiovascular events (MACE) was more heterogeneous and largely derived from small, methodologically limited studies. Overall, the available data support a pathophysiological link between IL-1–driven inflammation and plaque vulnerability but remain insufficient to establish robust prognostic utility or guide individualized perioperative risk stratification.

The main finding of this review is the recurrent association between upregulated IL-1–related cytokines, NLRP3 inflammasome components, and markers of innate immune activation with histological features of vulnerable carotid plaque. Several studies reported higher expression of NLRP3, IL-1–related mediators and associated inflammatory markers in plaques from symptomatic patients compared with asymptomatic individuals and in lesions with intraplaque haemorrhage, large lipid cores, or extensive macrophage infiltration [10,11,20]. These observations are consistent with experimental work showing that IL-1 signalling promotes leukocyte recruitment, extracellular matrix degradation, and necrotic core expansion, thereby driving the transition from stable to rupture-prone lesions [21]. Importantly, this association was observed despite substantial variability in sample processing and analytical methods, reinforcing the biological plausibility of NLRP3/IL-1-mediated plaque destabilization [10,11,20].

Beyond this central signal, several studies highlighted the broader inflammatory milieu that accompanies NLRP3/IL-1activation. Some cohorts demonstrated concurrent upregulation of downstream cytokines and chemokines, as well as markers of oxidative stress and macrophage polarization, suggesting that IL-1–driven pathways integrate into complex cytokine networks rather than acting in isolation [17,22]. Other investigations reported associations between inflammatory profiles and clinical presentation. Notably, symptomatic patients exhibited a much stronger systemic inflammatory signature, characterized by a highly coordinated network of circulating cytokines that remained elevated for months following the initial neurological event [15,17]. For instance, in a cohort of 46 patients, Stauss et al. demonstrated that symptomatic individuals had significantly higher plasma levels of IL-1β, IL-2, IL-3, IL-7, IFN-α2, and Fractalkine compared to asymptomatic patients, revealing a distinct inter-cytokine correlation network that persisted even at 90-day follow-up [15]. Similarly, Profumo et al. analyzed peripheral blood from 106 patients and found that the percentage of circulating cells expressing intracellular IL-1β, IL-6, and IFN-γ was significantly elevated in patients with a history of stroke compared to asymptomatic patients [17].

A smaller number of studies evaluated the relationship between NLRP3/IL-1 -related biomarkers and clinical outcomes after carotid endarterectomy, including perioperative events, restenosis, and long-term cardiovascular morbidity and mortality. For short-term outcomes, prospective evaluations indicated that elevated baseline and peak postoperative serum inflammatory markers strongly correlated with increased rates of 30-day major adverse cardiac and cerebrovascular events. In particular, Ahmed et al. reported that elevated baseline and peak postoperative serum IL-1β, IL-6, and CRP levels were associated with higher rates of early major adverse cardiac events, supporting a possible link between perioperative inflammatory activation and short-term postoperative risk [16]. Regarding long-term outcomes, large retrospective and prospective cohorts demonstrated that highly elevated intraplaque or peripheral blood IL-1β/NLRP3 expression significantly increased the risk of long-term composite MACE [13,20]. Within these cohorts, Marfella et al. demonstrated that patients with high intraplaque IL-1β burden had an increased risk of non-fatal stroke, non-fatal myocardial infarction, and all-cause mortality, whereas attenuation of this inflammatory pathway with PCSK9 inhibitors significantly reduced long-term events [13]. Furthermore, Xie et al. reported that increased PBMC expression of NLRP3 inflammasome components correlated with plaque instability and a greater incidence of composite cardiovascular and cerebrovascular events during follow-up, although most of these associations were not adjusted for key confounders [20]. Conversely, attenuation of this inflammatory pathway, such as through the administration of PCSK9 inhibitor monotherapy was linked to lower plaque NLRP3/caspase-1/IL-1β/TNF-α/NF-κB expression, higher SIRT3 and collagen content, and fewer markers of plaque instability, together with a substantially lower rate of long-term composite MACE. Taken together, these data suggest that targeted attenuation of NLRP3/IL-1related plaque inflammation may not only promote a more fibrotic, stable carotid phenotype but also confer meaningful protection against long-term cardiovascular events after CEA, although confirmation in randomized settings is still required [13]. Some reports suggested that higher inflammatory activity within the plaque or circulation may be associated with an increased risk of adverse events, but results were inconsistent, and only two studies used multivariable models to adjust for key confounders such as age, cardiovascular risk factors, and degree of stenosis.

This study faced many limitations worth noting. First, few articles were eligible for this systematic review, and the majority of those that were had small sample sizes without justification or power descriptions, which led to low precision in the obtained results. Moreover, there was substantial heterogeneity across studies in most baseline patient characteristics, study designs, and methodologies. Furthermore, few other short- and long-term outcomes were systematically assessed, which limited the evaluation of the association between NLRP3/IL-1–mediated inflammation and a broader range of clinically relevant endpoints. Most included studies were not designed to evaluate clinical outcomes after CEA. The majority were primarily plaque biology, biomarker, or mechanistic investigations in which CEA served as the tissue acquisition platform rather than the index intervention under study. Therefore, evidence regarding perioperative and long-term postoperative events remains sparse, and the findings presented in these tables should be interpreted accordingly.

This systematic review also highlights the need for additional large cohorts and randomized controlled trials with a primary aim of assessing the incidence and effect of NLRP3/IL-1-related inflammatory activity in patients undergoing carotid endarterectomy to validate the current findings and further uncover existing knowledge gaps. The potential impact of these pathways on plaque vulnerability and postoperative risk may ultimately predispose clinicians to adapt perioperative and long-term strategies for better management of this population. Nonetheless, more studies on the prevention and modulation of NLRP3/IL-1-mediated inflammation are crucial to avoid further harm to patients in the short and long term.

Interpretation of pyroptosis in endarterectomy tissue complements clinical outcomes and patient follow-up, as atherosclerotic plaque heterogeneity enables prediction of outcomes. The interplay between IL-1β, NLRP3, macrophages, and neutrophils drives inflammation and modulates wound healing in heterogeneous plaques in a context of concomitant angiogenesis and myofibroblast activity.

## 5. Conclusions

This systematic review establishes the NLRP3/IL-1 inflammasome axis as a crucial biological indicator of plaque vulnerability. Specifically, elevated local and systemic levels of these inflammatory biomarkers strongly correlate with histological features of plaque instability and a higher incidence of symptomatic cerebrovascular events, directly linking them to atherosclerotic progression. Nevertheless, significant methodological heterogeneity across current studies precludes their immediate integration into routine clinical practice and pathology. Future investigations must prioritize large-scale, prospective cohorts utilizing standardized protocols to determine their independent prognostic value following endarterectomy. Ultimately, validating this inflammatory pathway could significantly refine risk stratification and advance targeted therapies for carotid atherosclerosis.

## Figures and Tables

**Table 2:** Populations demographics and risk factors.

Author	Mean Age	Male n (%)	Arterial Hypertension n (%)	Dyslipidemia n (%)	Diabetes Mellitus n (%)	Smoking History n (%)	Coronary Artery Disease n (%)	Carotid Territory Symptoms n (%)
[8]	NA	NA	11 (55.0)	NA	5 (25.0)	14 (70.0)	NA	15 (75.0)
[9]	71.4	32 (78.0)	NA	NA	NA	NA	NA	18 (43.9)
[10]	74 (median)	34/41 in CAS cohort (83.0); 14/20 controls (70.0)	35 (60.3)	NA	17 (29.3)	8 (13.8)	NA	30 (51.7)
[11]	66.53	25 (83.3)	24 (80.0)	NA	15 (50.0)	13 (43.3)	NA	NA
[12]	69.3	132 (66.0)	153 (77.7)	NA	66 (33.5)	67 (34.0)	NA	105 (53.3)
[13]	NA	NA	NA	NA	NA	NA	NA	NA
[14]	NA	NA	NA	NA	NA	NA	NA	NA
[15]	NA	NA	NA	NA	NA	NA	NA	22 (47.8)
[16]	NA	NA	NA	NA	NA	NA	NA	NA
[17]	NA	NA	NA	NA	NA	NA	NA	NA
[18]	68.5	43 (75.4)	40 (70.2)	31 (54.4)	16 (28.1)	18 (31.6)	NA	23 (40.4)
[19]	NA	NA	NA	NA	NA	NA	NA	10 (50.0)
[20]	NA	NA	NA	NA	NA	NA	NA	NA
[21]	NA	NA	NA	NA	NA	NA	NA	NA
[22]	NA	NA	NA	NA	NA	NA	NA	NA
[23]	66.29	45 (69.2)	NA	NA	NA	NA	NA	NA

Legend: NA—unavailable data.

**Table 3:** Biomarkers measurements by study.

Author	Biomarker	Method	Exact Values/Expression Levels	Sample Source
[8]	IL-1β	Quantitative	Radial artery baseline: 14.1 ± 0.97 pg/mL; Jugular bulb 5 min post-declamping spiked to 35.7 ± 5.62 pg/mL (*p* < 0.01) in non-shunted patients.	Plasma
	PLA2, PGE2	Quantitative	Significantly increased in the jugular bulb in non-shunted patients following ischemia–reperfusion (*p* < 0.05).	Plasma
[9]	ST2L (IL-33 receptor)	Semiquantitative	Correlated with vulnerability; widely expressed in inflammatory plaque infiltrates.	Carotid plaque tissue
[10]	PTX3	Mixed (Q + SQ)	Local serum PTX3 significantly higher than systemic: 3.82 ± 0.45 ng/mL vs. 2.65 ± 0.30 ng/mL (*p* = 0.012).	Serum + Carotid plaque tissue
	IL-6	Mixed (Q + SQ)	Plaque positive area approx. 1%; significantly correlated with vulnerability (*p* < 0.05).	Serum + Carotid plaque tissue
	CRP	Mixed (Q + SQ)	Plaque positive area approx. 10%; localized heavily in macrophage-rich areas.	Serum + Carotid plaque tissue
	IL-10	Mixed (Q + SQ)	Expression was significantly decreased in vulnerable plaques (*p* < 0.05).	Serum + Carotid plaque tissue
	IL-1β, IFN-γ, MMP-9	Mixed (Q + SQ)	No significant differences detected between vulnerable and stable plaques or symptomatic/asymptomatic patients.	Serum + Carotid plaque tissue
[11]	IL-1β	Mixed (Q + SQ)	60% expression in unstable plaques vs. 10% in stable plaques (*p* < 0.05).	Carotid plaque tissue + Serum
	NLRP3	Semiquantitative	Expressed in 69% of unstable plaques vs. 20% of stable plaques (*p* < 0.05).	Carotid plaque tissue
	Caspase-1	Semiquantitative	30% higher relative expression in vulnerable lesions.	Carotid plaque tissue
	ASC	Semiquantitative	Significantly higher mRNA/protein expression in unstable plaques (*p* < 0.05).	Carotid plaque tissue
	IL-18	Mixed (Q + SQ)	Significantly elevated in the serum of symptomatic patients (*p* < 0.05).	Serum + Carotid plaque tissue
[12]	IL-1β, IFN-γ	Quantitative	Showed statistically significant fold-increases in elderly symptomatic plaques vs. asymptomatic (*p* < 0.05).	Carotid plaque tissue
	TNF-α	Quantitative	Showed no age-dependent difference (*p* > 0.05).	Carotid plaque tissue
[13]	IL-1β and NLRP3	Semiquantitative	IL-1β expression reduced by ~40% in PCSK9i treated patients; NLRP3 downstream effects significantly blunted (*p* < 0.01).	Carotid plaque tissue
[14]	IGF-1, IGFBP-1	Semiquantitative	Overexpressed in carotid plaques vs. controls; correlated with smooth muscle cell proliferation.	Carotid plaque tissue
[15]	IL-1β	Quantitative	Elevated plasma levels in symptomatic stenosis: 0.18 ± 0.05 pg/mL (*p* < 0.05 vs. asymptomatic).	Plasma
	CX3CL1	Quantitative	Elevated plasma levels in symptomatic stenosis: 418.2 ± 120.0 pg/mL.	Plasma
	IFN-α2, IL-2, IL-3, IL-7	Quantitative	All showed significantly higher plasma concentrations in symptomatic vs. asymptomatic patients (*p* < 0.05).	Plasma
[16]	IL-1, IL-6, ICAM-1, CRP	Mixed (Q + SQ)	Baseline and peak post-operative levels correlated strongly with Major Adverse Cardiac Events (MACE).	Serum
[17]	TNF-α, IFN-γ, IL-1β, IL-6, IL-8, IL-4, IL-10	Mixed (Q + SQ)	Higher intracellular cytokine expression in patients undergoing CEA versus non-CEA patients: TNF-α (*p* = 0.02), IFN-γ (*p* = 0.001), IL-1β (*p* = 0.04), IL-6 (*p* = 0.0003), IL-8 (*p* = 0.009), IL-4 (*p* = 0.0001), IL-10 (*p* = 0.001); additional elevations in patients with stenosis ≥70%, prior stroke, or amaurosis fugax	Peripheral blood mononuclear cells (PBMCs)
[18]	IFN-γ, IL-17, IL-22, IL-23	Quantitative	Highlighted distinct TH1, TH17, and TH22 lineage infiltrations directly inside the carotid atheroma.	
	IL-1β	Quantitative	mRNA: Significantly upregulated relative expression in plaques vs. controls (*p* < 0.05) Protein(IHC): Higher semi-quantitative scores in unstable plaques (scored ++ to +++) vs. stable plaques (scored + to ++).	
[19]	CD68, IL-1β, TNF-α, PTX-3, NF-κB, CRP, TGF-β, MMP-9	Quantitative	Higher density of macrophage-associated inflammatory markers localized specifically to rupture-prone plaque shoulders.	Carotid plaque tissue
[20]	NLRP3, IL-1β	Mixed (SQ + Q)	Unstable plaques (MPVRS > 4) showed significantly higher mRNA and protein expression of NLRP3 inflammasome components in PBMCs than stable plaques (MPVRS ≤ 4); plasma IL-1β and IL-18 were increased versus controls but did not differ significantly between unstable and stable plaque groups	Peripheral blood mononuclear cells (PBMCs)
[21]	MCP-1	Quantitative	Production decreased significantly with MyD88DN blockade (*p* = 0.000).	In vitro cell culture
	IL-8	Quantitative	Production decreased significantly with MyD88DN blockade (*p* = 0.006).	In vitro cell culture
	IL-6	Quantitative	Production decreased by 45.9% at 72 h when treated with IL-1 receptor antagonist (*p* = 0.009).	In vitro cell culture
	IL-6	Quantitative	Production decreased significantly with MyD88DN blockade (*p* = 0.002).	In vitro cell culture
	MMP-1	Quantitative	Production decreased significantly with MyD88DN blockade (*p* = 0.002).	In vitro cell culture
[22]	FerrylHb	Mixed (SQ + Q)	Oxidation hotspots definitively identified at β1Cys93, β1Cys112, and β2Cys112.	Carotid plaque tissue
	IL-1β	Mixed (SQ + Q)	Significantly upregulated at mRNA and protein levels in macrophages exposed to FerrylHb and highly expressed in complicated hemorrhagic plaques.	Carotid plaque tissue
	TNF-α	Mixed (SQ + Q)	Significantly upregulated at mRNA and protein levels in FerrylHb-exposed macrophages; highly expressed in complicated lesions.	Carotid plaque tissue
	HO-1 (Heme oxygenase-1), H-ferritin	Mixed (SQ + Q)	Highly upregulated in complicated plaques and in macrophages following FerrylHb internalization.	Carotid plaque tissue
[23]	CRP, fibrinogen, TNF-α, IL-1β, IL-6, IL-10	Semiquantitative	Increased pro-inflammatory markers correlated directly with ultrasound-confirmed plaque hypoechogenicity.	Serum

Legend: ASC—Apoptosis-associated speck-like protein containing a CARD; CD68—Cluster of Differentiation 68 (Macrophage marker); CRP—C-Reactive Protein; CX3CL1—Chemokine (C-X3-C motif) ligand 1 (Fractalkine); CXCR4—C-X-C chemokine receptor type 4; ELISA—Enzyme-Linked Immunosorbent Assay; FerrylHb—Ferrylhemoglobin; ICAM-1—Intercellular Adhesion Molecule 1; IFN-α2—Interferon alpha 2; IFN-γ—Interferon gamma; IGF-1—Insulin-like Growth Factor 1; IGFBP-1—Insulin-like Growth Factor Binding Protein 1; IHC—Immunohistochemistry; IL—Interleukin (e.g., IL-1β, IL-6, IL-8, IL-10, IL-18); MACE—Major Adverse Cardiac Events; MCP-1—Monocyte Chemoattractant Protein-1; MMP—Matrix Metalloproteinase (e.g., MMP-1, MMP-3, MMP-9); MPVRS—Modified Plaque Vulnerability Risk Score; MyD88DN—Myeloid Differentiation primary response 88 Dominant Negative; NF-κB: Nuclear Factor kappa-light-chain-enhancer of activated B cells; NLRP3—NOD-like receptor family pyrin domain containing 3; PCSK9i—Proprotein Convertase Subtilisin/Kexin type 9 inhibitors; PGE2—Prostaglandin E2; PLA2—Phospholipase A2; PTX3—Pentraxin 3; qPCR/RT-PCR—Quantitative/Reverse Transcription Polymerase Chain Reaction; ST2L—ST2 Ligand (Interleukin-1 receptor-like 1 Transmembrane Receptor); TGF-β—Transforming Growth Factor beta; TH1, TH17, TH22—T helper cell types 1, 17, and 22; TNF-α—Tumor Necrosis Factor alpha.

**Table 4:** Perioperative outcomes.

Author	Stroke 30 Days n (%)	Stroke/Death 30 Days n (%)	Death 30 Days n (%)	MI 30 Days n (%)	MACCE 30 Days n (%)	MACCE Definition	Post-Operative Adverse Events 30 Days n (%)	Restenosis n (%)
[8]	1 (5.0)	1 (5.0)	0 (0.0)	NA	NA	NA	Stroke 1 h after waking; urgent reoperation showed occluded endarterectomy site	NA
[9]	NA	NA	NA	NA	NA	NA	Not reported	NA
[10]	NA	NA	NA	NA	NA	NA	Not reported	NA
[11]	NA	NA	NA	NA	NA	NA	Not reported	NA
[12]	NA	NA	NA	NA	NA	NA	NA	NA
[13]	NA	NA	NA	NA	NA	NA	Not reported (plaque inflammation study; secondary endpoint was 2-year MACE)	NA
[14]	NA	NA	NA	NA	NA	NA	Not reported	NA
[15]	NA	NA	NA	NA	NA	NA	Not reported	NA
[16]	1 (1.3)	NA	NA	NA	13 (17.3)	Cardiac death, myocardial infarction, unstable angina	Troponin rise (n = 4), EKG changes (n = 4), troponin + EKG (n = 4), stroke (n = 1)	NA
[17]	NA	NA	NA	NA	NA	NA	Not reported	NA
[18]	NA	NA	NA	NA	NA	NA	Not reported	NA
[19]	NA	NA	NA	NA	NA	NA	Not reported	NA
[20]	NA	NA	NA	NA	NA	NA	Not reported for 30 days (study reports follow-up events)	NA
[21]	NA	NA	NA	NA	NA	NA	Not reported	NA
[22]	NA	NA	NA	NA	NA	NA	Not reported	NA
[23]	NA	NA	NA	NA	NA	NA	Not reported	NA

Legend: EKG—eletrocardiogram; MACCE—Major Adverse Cardiovascular and Cerebrovascular Events; NA—unavailable data.

**Table 5:** Long-term outcomes.

Author	Other Outcomes	Long-Term Outcomes	Long-Term Follow-Up Time	Long-Term MI n (%)	Long-Term Stroke n (%)	Long-Term MACE n (%)	Long-Term All-Cause Mortality n (%)
[8]	NA	Not reported	NA	NA	NA	NA	NA
[9]	NA	Not reported	NA	NA	NA	NA	NA
[10]	NA	Not reported (paper notes no ischemic events occurred between MR examinations and CAS)	NA	NA	NA	NA	NA
[11]	NA	NA	NA	NA	NA	NA	NA
[12]	NA	Not reported	NA	NA	NA	NA	NA
[13]	Events by group: 10/159 PCSK9i vs. 82/486 oLLD	Composite MACE (non-fatal MI, non-fatal stroke, all-cause mortality) assessed after CEA; secondary endpoint	678 ± 120 days (follow-up)	NA	NA	92 (14.3)	NA
[14]	NA	Not reported	NA	NA	NA	NA	NA
[15]	NA	No clinical events reported; biomarker follow-up only	90 days (biomarker follow-up sample)	NA	NA	NA	NA
[16]	NA	NA	NA	NA	NA	NA	NA
[17]	NA	Not reported	NA	NA	NA	NA	NA
[18]	NA	Not reported	NA	NA	NA	NA	NA
[19]	NA	Not reported	NA	NA	NA	NA	NA
[20]	Events: 8/38 (21.1%) unstable vs. 3/22 (13.6%) stable; 0/40 controls	Postoperative neurological/cardiovascular ischemic events (death, stroke, TIA, amaurosis fugax, angina, acute MI) assessed by phone/visit; composite events reported	Median 33.99 months (Kaplan–Meier); mean follow-up time not specified in extracted text	NA	NA	11 (18.3)	NA
[21]	NA	Not reported	NA	NA	NA	NA	NA
[22]	NA	Not reported	NA	NA	NA	NA	NA
[23]	NA	Not reported	NA	NA	NA	NA	NA

Legend: MACE—major adverse cardiovascular events; MI—myocardial infarction; NA—unavailable data.

## Data Availability

No new data were created or analyzed in this study.

## Supplementary Materials

The following supporting information can be downloaded at https://www.mdpi.com/article/10.3390/medsci14020280/s1, Table S1: Search query—key words; Table S2: Covariates used in the adjusted models; Table S3: Pre-operative characteristics.
