# Supplementary material for: NLRP3 Inflammasome and IL-1–Mediated Inflammation in Human Carotid Atherosclerosis: A Systematic Review of Endarterectomy-Based Evidence
Source: Med Sci (Basel). 2026 May 31;14(2):280. doi: 10.3390/medsci14020280 (PMC13304386; doi:10.3390/medsci14020280)
Supplement: Supplementary file 1 [file medsci-14-00280-s001.zip › medsci-4278234-supplementary.pdf]

Supplementary Table 1 – Search query – key words

**Bibliographic source**	**Search term**	**No of reports**
Pubmed	(("NLRP3" OR "Nuclear oligomerization domain-Leucine rich repeat- and pyrin domain-containing protein 3" OR "NOD-LRR- and pyrin domain-containing protein 3" OR "NLR family, pyrin domain containing 3 protein, human" OR "cryopyrin" OR "NLRP3 inflammasome") OR ("Interleukin-1" OR "IL-1" OR "interleukin 1")) AND ("Endarterectomy, Carotid" OR "carotid endarterectomy" OR CEA)	108
ISI	(("NLRP3" OR "Nuclear oligomerization domain-Leucine rich repeat- and pyrin domain-containing protein 3" OR "NOD-LRR- and pyrin domain-containing protein 3" OR "NLR family, pyrin domain containing 3 protein, human" OR "cryopyrin" OR "NLRP3 inflammasome") OR ("Interleukin-1" OR "IL-1" OR "interleukin 1")) AND ("Endarterectomy, Carotid" OR "carotid endarterectomy" OR CEA)	127
SCOPUS	(("NLRP3" OR "Nuclear oligomerization domain-Leucine rich repeat- and pyrin domain-containing protein 3" OR "NOD-LRR- and pyrin domain-containing protein 3" OR "NLR family, pyrin domain containing 3 protein, human" OR "cryopyrin" OR "NLRP3 inflammasome") OR ("Interleukin-1" OR "IL-1" OR "interleukin 1")) AND ("Endarterectomy, Carotid" OR "carotid endarterectomy" OR CEA)	302

Supplementary Table 2. Covariates used in the adjusted models

**Author**	Covariables used in adjusted models
Ahmed 2025 [10]	NA (comparisons between MACE vs non-MACE using t-tests; no multivariable adjusted model reported).
Potor 2021 [11]	NA (mechanistic plaque/RNA-seq study; no adjusted clinical model).
Puz 2013 [12]	NA (comparative analyses; no multivariable adjusted model reported).
Grufman 2014 [13]	ANCOVA (adjusted for gender, BMI, diabetes, hypertension, smoking, creatinine, triglycerides, LDL, HDL, and medication use (statins, beta-blockers, ACE inhibitors, ARB, ASA, other antiplatelet agents)).
Marfella 2023 [14]	Cox proportional hazards regression (adjusted for age, sex, BMI, hypertension, LDL-cholesterol, HbA1c, creatinine levels).
Wang 2012 [15]	NA / not reported
Monaco 2009 [16]	NA (in vitro plaque cell culture study; no adjusted model).
Profumo 2008 [17]	No clearly reported adjusted multivariable model; mainly univariable/non-parametric group comparisons, with limited discussion of possible confounding by baseline characteristics.
Pärsson 2000 [18]	NA (no adjusted model reported)
Hakimi 2013 [19]	NA (histopathological/IHC scoring; no adjusted model).
Marzullo 2016 [20]	NA (mechanistic/ex vivo study; no adjusted model)
Stauss 2020 [21]	Binary logistic regression (Grade of stenosis, ESRS (Essen Stroke Risk Score), antithrombotic pretreatment, statin pretreatment).
Xie 2023 [22]	NA (no multivariable adjusted model described; group comparisons stable vs vulnerable plaques).
Shi 2015 [23]	NA (descriptive comparison; no adjusted model reported)
Shindo 2014 [24]	NA (imaging/biomarker observational; no adjusted model reported)
Oliveira 2013 [25]	NA (gene expression/IHC/flow cytometry; no adjusted model).

BMI – body mass index; CRP – C-reactive protein; FT3 - free triiodothyronine; FT4 - free thyroxine; HDL – high-density lipoproteins; LDL – low-density lipoproteins; TNF-α – tumor necrosis factor alpha; TSH – thyroid-stimulating hormone; TSHI – thyroid-stimulating hormone index; TT4RI - thyrotroph T4 resistance index; TFQI -thyroid feedback quantile-based index.

Supplementary Table 3 – Pre-operative characteristics

**Author**	Contralateral stenosis n(%)	Antiplatelet therapy n(%)	Anticoagulation n(%)	General anesthesia n(%)	Regional anesthesia n(%)
Ahmed et al. [10]	NA	NA	NA	NA	NA
Potor et al. [11]	NA	NA	NA	NA	NA
Puz et al. [12]	NA	NA	NA	NA	NA
Grufman et al. [13]	NA	NA	NA	NA	NA
Marfella et al. [14]	NA	NA	NA	NA	NA
Wang et al. [15]	NA	NA	NA	NA	NA
Monaco et al. [16]	NA	NA	NA	NA	NA
Profumo et al. [17]	CEA: 45/67 (67.2); non-CEA: 36/39 (92.3)	106 (100) aspirin	NA	NA	NA
Pärsson et al. [18]	NA	NA	NA	NA	NA
Hakimi et al. [19]	NA	NA	NA	NA	NA
Marzullo et al. [20]	NA	NA	NA	NA	NA
Stauss et al. [21]	NA	NA	NA	NA	NA
Xie et al.	NA	NA	NA	NA	NA

[22]					
Shi et al. [23]	NA	NA	NA	NA	NA
Shindo et al. [24]	NA	NA	NA	NA	NA
Oliveira et al. [25]	NA	42 (73.7)	NA	NA	NA

Legend: NA – unavailable data
